# The *S*-Gene *YUC6* Pleiotropically Determines Male Mating Type and Pollen Size in Heterostylous *Turnera* (Passifloraceae): A Novel Neofunctionalization of the YUCCA Gene Family

**DOI:** 10.3390/plants11192640

**Published:** 2022-10-08

**Authors:** Paige M. Henning, Joel S. Shore, Andrew G. McCubbin

**Affiliations:** 1 School of Biological Sciences, Washington State University, Pullman, WA 99164-4236, USA; 2 Department of Biology, York University, Toronto, ON M3J 1P3, Canada

**Keywords:** distyly, *Turnera*, heterostyly, self-incompatibility, mating type, *YUC6*, auxin

## Abstract

In heterostylous, self-incompatible *Turnera* species, a member of the *YUCCA* gene family, *YUC6*, resides at the *S*-locus and has been hypothesized to determine the male mating type. *YUCCA* gene family members synthesize the auxin, indole-3-acetic acid, via a two-step process involving the *TAA* gene family. Consequently, it has been speculated that differences in auxin concentration in developing anthers are the biochemical basis underlying the male mating type. Here, we provide empirical evidence that supports this hypothesis. Using a transgenic knockdown approach, we show that *YUC6* acts pleiotropically to control both the male physiological mating type and pollen size, but not the filament length dimorphism associated with heterostyly in *Turnera*. Using qPCR to assess *YUC6* expression in different transgenic lines, we demonstrate that the level of *YUC6* knockdown correlates with the degree of change observed in the male mating type. Further assessment of *YUC6* expression through anther development, in the knockdown lines, suggests that the male mating type is irreversibly determined during a specific developmental window prior to microsporogenesis, which is consistent with the genetically sporophytic nature of this self-incompatibility system. These results represent the first gene controlling male mating type to be characterized in any species with heterostyly.

## 1. Introduction

**Figure 1:**
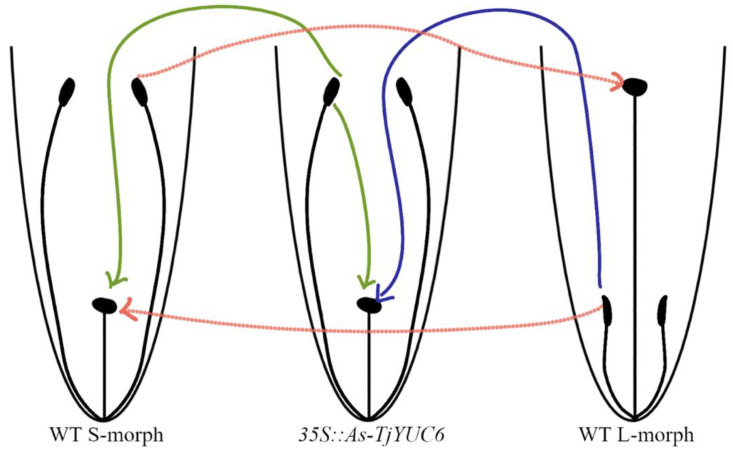
Model of the most extreme observed change in male mating type but retention of female mating type in the knockdown *TjYUC6* lines. Green lines represent successful pollination from the transgenic lines with self and WT lines. Blue line represents successful pollinations from the WT lines with transgenic lines. Dotted red lines represent WT pollinations.

Distyly is an angiosperm reproductive system that confers self- (and intramorphic) incompatibility (SI). Distylous plants exhibit two floral morphs, the short- (S-) and long-styled (L-) morphs, named for their reciprocal positioning of stigmas and anthers (Figure 1) but also differing in other morphological characters, such as the length of the stigmatic papillae, pollen size, and biochemical mating type [1]. Distyly has evolved independently in at least 28 families of angiosperms with strong consistencies in phenotypes but not in the genes that control the dimorphisms, suggesting that it represents an intriguing example of convergent evolution [1].

Early Mendelian geneticists inferred from segregation data that the presence of distyly was likely a result of a supergene complex, for which the S-morph was heterozygous and the L-morph homozygous recessive [2]. This supergene would later be called the self-incompatibility (or *S*-) locus [3]. The *S*-loci that govern distyly likely evolved to maintain the complex of morphological traits and SI mating types, as recombination does not occur within supergenes [4,5]. Recent molecular investigations have supported the supergene hypothesis [6,7,8,9] but necessitate a revision to the model. Analyses of the *S*-loci of *Primula* [7], *Turnera* [9], *Fagopyrum* [8], and *Linum* [6] have shown, or suggest, that the S-morph is hemizygous rather than heterozygous for the *S*-locus, as the L-morph lacks allelic counterparts to the *S*-genes that reside at the *S*-locus. As all pollen of the hemizygous S-morph carries the same mating type, distyly is a form of sporophytic SI (SSI) (at least for S-morph). In SSI systems, the diploid genotype of the sporophytic tissue, within which gamete producing gametophytes (pollen and ovules) are produced, determines the mating type of the haploid gametophytes [10].

The identity of the genes that reside at the *S*-loci that control heterostyly have been reported for several species. In *Fagopyrum*, female morphology (style length) is determined by *S-ELF3*, a homolog of *Arabidopsis ELF3* [11]. In *Arabidopsis*, *ELF3*, in conjunction with *ELF4*, acts to repress the transcription of circadian clock-related genes indirectly inhibiting hypocotyl elongation and flowering time, consistent with the *Fagopyrum* homolog acting to reduce style length [12].

In *Primula*, the entire *S*-locus has been characterized and is composed of five *S*-genes, *CCM*, *GLO2*, *CYP734A50*, *PUM*, and *KFB*, each apparently having been incorporated into the *S*-locus via independent duplication events [13,14,15]. Empirical evidence has demonstrated that *CYP734A50* controls female morphology and the female mating type [16,17]. The mechanism by which *CYP734A50* affects the female phenotype is by inactivating brassinosteroid (BR) to decrease active levels of this hormone in S-morph pistils [16]. *GLO2* controls anther height and it is involved in corolla tube elongation below the stamen (to which the short filament that holds the anther is attached in this species); its presence positions the stamen higher in the S-morph [18]. The roles of *CCM*, *PUM*, and *KFB* in distyly have not been elucidated. 

In *Turnera*, again, the entire *S*-locus has been characterized and contains just three *S*-genes: *BAHD*, *S-protein homolog 1* (*SPH1*), and *YUCCA 6* (*YUC6*) [9]. *BAHD*, a member of the *BAHD* acyltransferase family, pleiotropically controls both female morphology and the female mating type [19,20]. Interestingly, like *Primula CYP734A50*, *Turnera BAHD* establishes female phenotypes by inactivating BR in S-morph pistils, illustrating a common molecular basis behind pistil phenotypes in these two genera [20,21]. *SPH1* is predicted to be involved in filament elongation; this gene is not expressed in short homostyle mutants [9]. The mechanism by which *SPH1* elicits cell elongation is unknown. The large *SPH* gene family encodes small signaling peptides, but the only characterized member is the *Papaver rhoeas* pistil incompatibility gene *PrsS* [22,23]; upon recognition by a second *S*-locus gene *PrsP*, the receptor, which is expressed in pollen, *PrsS* elicits a Ca^2+^-dependent SI response that results in the death of the pollen/pollen tube [24,25]. Finally, *YUC6* is hypothesized to determine the male mating type [9]. Based on homology, *YUC6* is predicted to be a member of the *YUCCA* gene family of flavin-containing monooxygenases, which are involved in synthesizing the plant hormone auxin, specifically indole-3-acetic acid (IAA) [9]; previous RNAseq analysis identified several auxin-related genes as differentially expressed between the anthers of two floral morphs of distylous *T. subulata* [21]. 

In addition to the identification of S-genes, work in Fagopyrum [26], Primula [27,28,29], Turnera [21,30,31], and also Lithospermum [32] suggest that genes outside of the *S*-loci are likely regulated by the *S*-gene products and involved in various aspects of distyly. Hence, the molecular basis of distyly likely involves a cascade of changes in transcription to produce the overall phenotypes observed. 

While the *S*-genes that have been identified in the three species characterized to date are different, it is becoming evident that between these species, at least, there are similarities in the mechanisms, by which female morphology and mating type are determined, i.e., the alteration of active BR levels to determine the female phenotype. Commonality in the means of obtaining a complex phenotype, such as distyly, may not be surprising, as many cases of convergent evolution share the alteration of one molecular or cellular process [33,34]. It has been hypothesized that PHYTOCHROME INTERACTING FACTOR (PIF) signaling hubs are a potential convergence point for the different mechanisms that have evolved to generate heterostyly [21]. Furthering our understanding of the molecular basis of distyly across genera will expand our understanding of the nature of the system and potential convergence.

Given the putative biochemical function of *YUC6*, we hypothesize that it determines the male mating type in distylous *Turnera* through its impact on auxin production. While there are several synthesis pathways and different chemical forms of auxin, the main auxin biosynthesis pathway is the tryptophan-dependent pathway [35]. This pathway generates indole-3-acetic acid (IAA) from tryptophan (TRP) in a two-step process; members of the *TAA* gene family convert TRP to indole-3-pyruvic acid (IPA) and, subsequently, *YUCCA* family members convert IPA to IAA [36]. The tryptophan-dependent pathway is likely ancestral to plants, as it has been identified in charophytes [37]. 

Auxin is one of the oldest phytohormones, as it is found in all lineages of green plants [38]. Auxin signaling appears to have originated in the charophytes and may have evolved in the last common ancestor of this group [38,39,40]. Interestingly, auxin is found outside of the plant kingdom; fungi and bacteria can synthesize and respond to auxin, likely as a result of horizontal gene transfer [37]. In angiosperms, auxin is involved in all major developmental processes [41,42], reproduction [43,44], biotic [45] and abiotic [46] stress responses, and communication with bacteria [41,47].

In *Arabidopsis thaliana*, a subclade (IIB) of the *YUCCA* family, is essential for reproduction [48]. There are four members in this subclade: *AtYUC1*, *AtYUC2*, *AtYUC4*, and *AtYUC6*; quadruple knockout mutants of this subclade show extreme floral defects, producing few to no flowers, when flowers are produced they are small, cylindrical, and sometimes lack organs [48]. It should be noted that these members also play roles outside of floral development, including embryogenesis, leaf and vascular development, and stress responses [46,49,50]. *AtYUC1* and *AtYUC4* play prominent roles in floral development, with double mutants negatively affecting the entire flower [48,51]. In addition, *AtYUC4* initiates gynoecium formation [52]. A splice variant of *AtYUC4* (*AtYUC4.2*) is flower specific [53]; the role of this splice variation is unknown, but it has been suggested that the alternative splicing may dictate subcellular localization [53]. The role of *AtYUC1* alone in floral development has not been assessed to our knowledge. 

*AtYUC2* and *AtYUC6* play critical roles in pollen and stamen development, and pollen germination in *A. thaliana*. Double mutants exhibit smaller stamens [48,54], and do not produce pollen [54]. *AtYUC6* mutants exhibit decreased anther dehiscence and pollen germination [55]. Interestingly, overexpression of *AtYUC6* results in decreased seed production [56]. Additionally, IAA synthesized by *AtYUC2* and *AtYUC6* in the tapetum is transported from the anther to the filaments to initiate filament elongation [54,55]. 

Here, we present empirical evidence that supports *YUC6* being the male mating-type determinant factor in distylous *Turnera joelii*. In addition, we show that *YUC6* is involved in pollen size dimorphism. Our results suggest that *YUC6* in distylous *Turnera* species have undergone significant evolutionary changes in gene expression and regulation to fulfill their role as the male mating-type determinant factor. 

## 2. Results

### 2.1. Knockdown of TjYUC6 in T. joelii

*YUC6* has been hypothesized to determine male mating type in distylous members of Turnera [9]. If this hypothesis is correct, the successful knockdown of *T. joelii YUC6* (*Tj YUC6*) in the S-morph is predicted to result in an “L-morph” male mating type while the “S-morph” female mating type would remain unchanged; consequently, the resulting transformant would be both self-compatible and incompatible when crossed as pollen parent with L-morph plants (Figure 1). 

To empirically test this hypothesis, S-morph tissue of *T. joelii* was transformed with *35S::Antisense-TjYUC6* (*35S::AsTjYUC6*) (Figures S1 and S2) and stable transgenic plants regenerated. *YUC6* is expressed in the stamen, it was unknown if the *35S* promoter would drive expression in the stamen of *T. joelii* at the outset of this experiment. This promoter is not expressed in the anthers or pollen of *Arabidopsis thaliana* [57], but it has been reported to be expressed in the anthers [58,59] and pollen [60,61] of a number of other plant species. The pollen mating type in heterostyly is genetically sporophytically encoded, i.e., it is determined by the diploid genotype of the mother plant. This can be achieved in several ways, either through the secretion of a factor by the mixed haploid genotype pollen to affect their phenotype, or the secretion of a factor by the diploid maternal anther cells (the pollen nourishing tapetum) [62]. The *35S* promoter was selected in the absence of data for the activity of any other promoter in *Turnera*, in either germline or sporophytic anther cells, as it was readily available. To optimize the chance of the knockdown construct being specific to *YUC6*, *YUC6* was aligned with the other YUC family members (Figure S3) and a region chosen based on it having relatively low homology to other isoforms identified (see Table S4 for GenBank Accession numbers). The resulting transformants were screened using controlled pollinations to test the predictions above (Figure 1).

To confirm that transformants exhibiting an SC phenotype were SC specifically, as a result of modification of the male mating type (i.e., the female mating type was not affected), we crossed the transgenic lines reciprocally to WT S- and L-morph plants. Once an altered male mating type was established, transgenic lines were assayed to confirm the presence of the *35S:AsTjYUC* insert (Figure S4).

**Figure 2:**
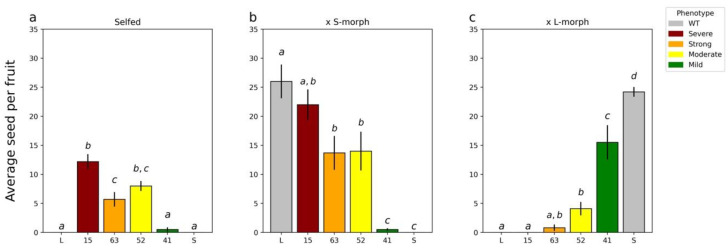
Seed set of T0 plants when self-pollinated and when acting as the pollen donor crossed with WT L- and S-morph plants. (**a**), self-pollination of T_0_ plants; (**b**), T_0_ plants crossed as male parent to wild type S-morph; (**c**), T_0_ plants crossed as male parent to L-morph plants. For all analyses means sharing the same letter are not significantly different following a single factor ANOVA and Tukey’s test. For seed set data, the square root transformation was applied prior to conducting the analysis of variance. Each cross was analyzed independently of the other crosses allowing for comparison of performance within a class. When selfed F_5,54_ = 53.2, *p* < 0.0001; when crossed with S-morph (×S) F_5,54_ = 23.9, *p* < 0.0001; and when crossed with L-morph (×L) F_5,54_ = 43.7, *p* < 0.0001. Error bars represent the standard error. N = 10 pollinations.

Four lines showed a change in the male mating type (Figure 2a–c). None of these lines showed a change in the female mating type (data not shown). T_0_ line 15 showed the strongest pollen parent phenotype; when crossed with WT, S-morph plants’ seed set was not significantly different from those performed with the L-morph, but it was significantly different from those with the S-morph (Figure 2b,c). Line 63 showed the second strongest phenotype; when crossed with the L-morph, again seed set was not significantly different from pollinations with L-morph pollen but was significantly different from those with S-morph pollen (Figure 2b). Line 52 showed an intermediate phenotype; in all crosses, seed set was significantly different from both morphs (Figure 2a–c). Finally, line 41 showed the weakest phenotype, setting the smallest number of seed when crossed with the S-morph and when self-pollinated (Figure 2a,b).

### 2.2. RT-qPCR Analysis of YUC6 Expression in Knockdown Lines

The ability of the *35S* promoter to drive expression in *T. joelii* anthers was confirmed by qPCR. Choosing T_0_ line 15, the line with the strongest phenotype, qPCR was performed with a forward primer designed to the transcribed end of the *35S* promoter and reverse primer within the AS-YUC6 sequence to specifically amplify the transgene product. Transgene expression was compared with that of the house keeping gene ß-tubulin. Transgene expression was confirmed in young and mature anthers and pollen samples (Figure S5), with expression levels (represented as difference in the cycle threshold of detection [∆CT]) being similar relative to ß-tubulin in young anther and pollen (~2 ∆CT), and somewhat lower in mature anthers (~5 ∆CT).

**Figure 3:**
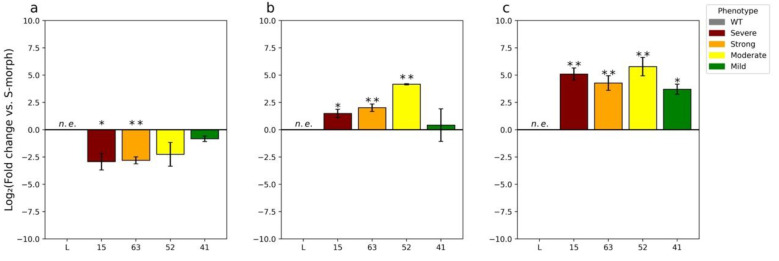
RT-qPCR analysis of the expression of TjYUC6 in T_0_ lines compared to WT S-morph during: early floral development (**a**), late floral development (**b**), and the pollen from dehisced anthers (**c**). N = 3 biological replicates, n.e. = not expressed, * = *p*-value < 0.05, and ** = *p*-value < 0.01. *p*-values determined by Student’s *t*-test comparing the ∆CT of the respective transgenic line with that of the WT S-morph. Error bars represent the standard error of the fold change. Values represent the log2 (fold change).

To determine if the observed changes in the male mating type were due to the knockdown of endogenous *TjYUC6*, we assessed *TjYUC6* mRNA levels by RT-qPCR (Figure 3a–c). The RT-qPCR graphs shown in Figure 3 compare *TjYUC6* mRNA levels in the anthers of the transformants with those in the anthers of WT S-morph. We compared mRNA levels at “young” (4–6 mm buds) and “mature” (13–15 mm buds) stages of development, as these stages were used previously in RNAseq analysis [21]. For the sake of clarity, these stages are referred to as young and mature anthers for the entirety of this manuscript. In the young anthers, a change in *TjYUC6* level was found in all four initial transformants (Figure 3a). The two lines with more robust phenotypes (i.e., substantial seed set on self-pollination, seed set in cross to S-morph, and reduced or no seed set in cross to L-morph) showed statistically significant reductions in *TjYUC6* mRNA when compared to the S-morph, as determined by a Student’s t-test. Overall, the levels of *TjYUC6* knockdown relative to the S-morph correlated with the strength of phenotypic change.

Somewhat surprisingly, all *35S:AsTjYUC6* lines showed an increase in *TjYUC6* mRNA levels (relative to the S-morph) at the mature anther stage of development (Figure 3b) and an even greater increase in pollen (Figure 3c). We hypothesize that this reflects a physiological feedback response to reduced auxin levels in the early stages of anther development, as reduced auxin levels stimulate the expression of YUC6 in Arabidopsis [62]. Previously, we had shown that *YUC6* expression decreases through development in S-morph anthers [9]. The early peak and subsequent drop in expression may be because the mating type is established early in development. To determine if our mature T_0_s are reaching the peak “WT” young anther levels, we compared the levels of *YUC6* expression in the mature anthers of the T_0_ with WT young anther (Figure S6). The majority of our T_0_ lines show lower expression levels than the WT S-morph young anthers. T_0_-52 does show levels similar to WT young anther; a potential explanation for this finding is discussed below.

As noted above, in the wild-type S-morph *TjYUC6*, expression levels decrease through floral development. To determine if the *35S:AsTjYUC6* lines were acting in a similar manner, we compared the mRNA levels of *TjYUC6* within lines (supplemental Figure S5a,b). When comparing WT S-morph mature anthers and pollen with the young anthers of the WT S-morph, we observed the previously reported decrease in *TjYUC6* mRNA levels (Figure S7a,b). Interestingly, in contrast T_0_-15, 52, and 63 all showed an increase in *TjYUC6* mRNA throughout development (Figure S7a,b). The *35S:AsTjYUC6* line with the weakest knockdown and phenotype, T_0_-41, showed a decrease in *TjYUC6* expression between young and mature stages (Figure S5a), but it did exhibit an increased expression in pollen relative to young anthers (Figure S7b). The identification of the *TjYUC6* transcript in pollen was surprising. We compared the expression levels of *TjYUC6* in the pollen to that of the mature stamen (Figure S7c). We see a higher expression of *TjYUC6* in the WT S-morph pollen than mature stamen, as well as in the T_0_ lines; this may suggest that at least some of the “expression” of *TjYUC6* in the stamen is caused by the expression or sequestering of transcript in the developing pollen. If this is the case, WT pollen (not carrying the transgene) within the T_0_ lines may be complicating the results of our RT-qPCR analysis; further analysis is required to investigate this possibility.

### 2.3. Additional Characterization of YUC6 Knockdown Lines 

**Figure 4:**
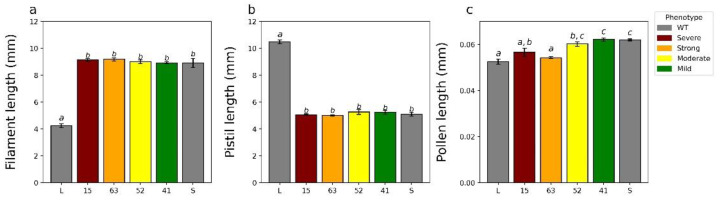
Quantification of the relevant floral characteristics of the T_0_ (**a**–**c**). For all analyses, means sharing the same letter are not significantly different following a single factor ANOVA and Tukey’s test. a, filament length; b, pistil length; c, pollen length. Error bars represent the standard error. Length of the pistils of three flowers (**a**) F_5,12_ = 277.5, *p* < 0.0001, N = 9 pistils, 3/flower; length of the filaments of three flowers (**b**) F_5,12_ = 120.3, *p* < 0.0001, N = 15 filaments, 5/flower; and mean length of pollen grains from three flowers (**c**) F_5,12_ = 17.1, *p* < 0.0001, N = 300 pollen grains, 100/flower.

**Figure 5:**
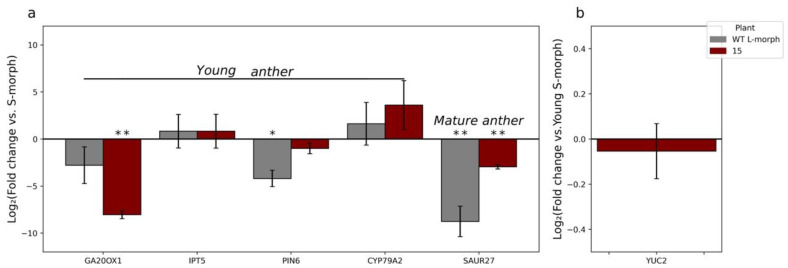
RT-qPCR analysis of previously identified differentially expressed auxin-related genes in T_0_-15. * = *p*-value < 0.05, and ** = *p*-value < 0.01. *p*-values determined by Student’s *t*-test comparing the ∆CT of T_0_-15 or WT L-morph anthers, with that determined for WT S-morph anthers, at the stages as labelled. Error bars represent the standard error of the fold change. Values represent the log2(fold change). Note, (**b**)’s scale is extremely small compared to (**a**)’s scale to better represent the miniscule and insignificant difference in expression.

To determine if *TjYUC6* influences morphological aspects of distyly, we measured pistil, filament, and pollen length in the *35S:AsTjYUC6* lines and the WT plants (Figure 4a–c). No significant difference in pistil or filament lengths was observed between the *35S:AsTjYUC6* lines relative to the WT S-morph (Figure 4a,b). However, there were significant differences in the pollen size of T_0_-15 and T_0_-63 (the lines with the most robust mating type change) compared to the S-morph, but not the L-morph (Figure 4c). As lines T_0_-52 and T_0_-49 did exhibit some change in the mating type, this observation suggests that there may be different auxin threshold levels for determining the mating type versus pollen size. Vegetative morphological features did not differ between the *35S:AsTjYUC6* lines and the WT S-morph (Figure S8), which is consistent with our antisense construct specifically targeting *YUC6* (which is only expressed in anthers), and not affecting other YUC isoforms (which are likely expressed in other plant tissues, similar to YUC homologs in *Arabidopsis* [49,50], though the expression patterns of *T. joelii YUC* family members are unknown at this time). RT-qPCR analysis of *YUC2* (Figure 5b), which is expressed in the young anther of *T. subulata*, did not show a change in expression when comparing our strongest line T_0_-15 to the S-morph.

To determine if changes in *YUC6* expression altered the expression of previously identified putative-related/regulated genes *GA20OX1*, *IPT5*, *PIN6*, *CYP79A2*, *SAUR27*, and *YUC2* [21], we compared the expression of the genes in our strongest line T_0_-15 with the expression of these genes in WT L-morph anthers, each relative to those observed in S-morph anthers. A list of these genes, and their relation to auxin can be found in Table S2. Overall, the genes quantified showed expression trends like that of L-morph anthers, though to varying degrees (Figure 5a). These results are consistent with knocking down the expression of *TjYUC6* influencing the expression of auxin-related genes. When comparing expression of YUC2 (which functions redundantly with *YUC6* in *Arabidopsis*) between T_0_-15 and the WT S-morph, no significant change was observed, which is consistent with our knockdown construct being specific to the YUC6 isoform (Figure 5b).

In distylous *Turnera*, pistil and filament dimorphisms are not established until mid to late floral development. To determine if pollen dimorphisms follow the trend of other morphological dimorphisms, we dissected anthers from both morphs during the young and mature stages of development and compared the pollen size to that of dehydrated pollen. Individual microspores (rather than pre-meiotic, meiotic cells, or tetrads) were identified in the stage of anther development that we have termed “young” (from 4–6 mm buds) in both morphs, suggesting that microsporogenesis had completed by this stage (Figure S9a–h). The size of the microspores/pollen did not start to diverge between morphs until after the young anther stage, but once initiated, divergence continued to increase through to pollen maturity (Figure S9a). Representative images of the pollen can be found in Figure S9b–g. 

The RT-qPCR results coupled with the phenotypes of the transgenic plants, and the presence of released microspores in young anthers, suggest that the male mating type is established and becomes fixed during a developmental window early in anther development at, or before, the young anther stage, presumably by altering auxin concentration. Increased *TjYUC6* expression observed at the mature anthers stage in transgenic lines (Figure 3) did not correlate with the severity of change in the male mating type. In contrast, the data suggest that pollen size dimorphisms are initiated after the young anthers stage and the possibility that they can be modified by *YUC6* expression later in development.

T_0_ lines 15 and 63 both exhibited small (L-morph) pollen size. Both also exhibited a substantial (~2.5 log_2_ fold change) reduction in *YUC6* expression in young anthers, and increased *YUC6* expression in mature anthers (Figure 2 and Figure 3a,b). However, despite being increased relative to the mature anthers of the S-morph, the expression levels reached were still significantly lower than those attained in S-morph young anthers (supplementary Figure S6). We hypothesize that there is a threshold level of *YUC6* expression needed to initiate cell expansion (leading to increased pollen size), which is reached in young anthers, but this was not reached in T_0_ lines 15 and 63. Extending this hypothesis to T_0_ lines 52 and 41, which exhibited a weaker change in the mating type and retained the large pollen size of the S-morph (Figure 2 and Figure 4c), in T_0_ line 52, *YUC6* expression in mature anthers was statistically equal to the level of *YUC6* expression in WT young anthers (Figure S6), and so reached the hypothesized threshold needed for S-morph pollen size. It is also necessary to infer that increased *YUC6* expression later in development can “rescue” large pollen size to explain this phenotype. T_0_ line 41 never surpassed the *YUC6* expression level in WT young anthers, but it only exhibited a small knockdown of *YUC6* at the young anther stage relative to the other lines (Figure 3a); we suggest that in this line the hypothesized threshold is still attained at the young anther stage.

### 2.4. Characterizing the T_1_ Generation

Seeking to determine if the change in the pollen mating type segregated with the transgene, T_0_-15 was selected to cross as the pollen parent with a WT S-morph individual to generate a T_1_ generation. T_0_-15 was chosen, as it exhibited the greatest decrease in *TjYUC6* mRNA levels and change in the mating type. 

**Figure 6:**
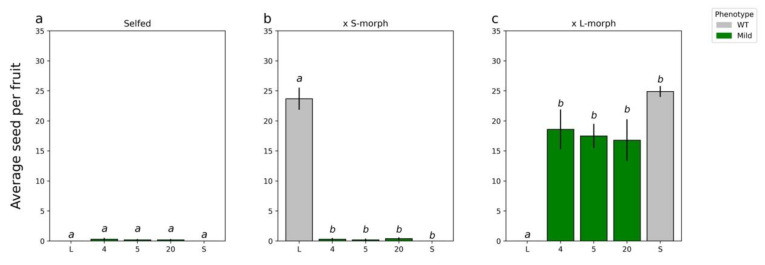
Seed set of the T_1_ when self-pollinated and when acting as the pollen donor when crossed with WT L- and S-morph. (**a**), self-pollination of T_0_ plants; (**b**), T_0_ plants crossed as male parent to wild type S-morph; (**c**), T_0_ plants crossed as male parent to L-morph plants. For all analyses, means sharing the same letter are not significantly different following a single factor ANOVA and Tukey’s test. For seed set data, the square root transformation was applied prior to conducting the analysis of variance. Each cross was analyzed independently of the other crosses allowing for comparison of performance within a class. When selfed F_5,54_ = 1.64 n.s., *p* > 0.18; when crossed with S-morph (×S) F_4,45_ = 249.0, *p* < 0.0001; and when crossed with L-morph (×L) F_4,45_ = 24.7, *p* < 0.0001. Error bars represent the standard error. N = 10 crosses.

**Figure 7:**
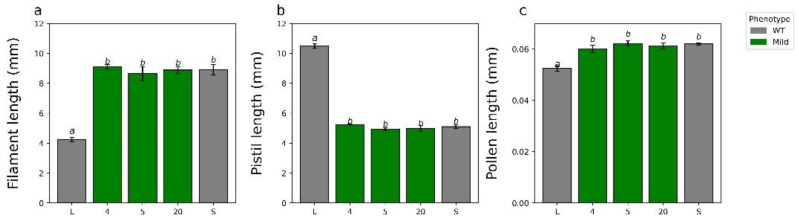
Quantification of the relevant floral characteristics of the T1 lines. (**a**), filament length; (**b**), pistil length; (**c**), pollen length. For all analyses, means sharing the same letter are not significantly different from each other following a single factor ANOVA and Tukey’s test. Error bars represent the standard error. Length of the pistils of three flowers (**a**) F_4,10_ = 314.5, *p* < 0.0001, N = 9 pistils, 3/flower; length of the filaments of three flowers (**b**) F_4,10_ = 48.8.5, *p* < 0.0001, N = 15 filaments, 5/flower; and mean length of pollen grains from three flowers (**c**) F_4,10_ = 14.6, *p* < 0.0004, N = 300 pollen grains, 100/flower.

T_1_ plants carrying *35S:AsTjYUC6* were raised from seed from an S-morph (female parent) × T_0_-15 (male parent) cross. A ratio of 16 S-morph: 7 L-morph was obtained and is not significantly different from a standard Mendelian 3:1 ratio (*p* = 0.36). Of the 16 progeny that were S-morph, 5 carried the *35S:AsTjYUC6* transgene and 11 did not, suggesting the presence of a single transgene that was not inserted on the chromosome bearing the *S*-locus. Three T_1_ S-morph progeny carrying *35S:AsTjYUC6* (Supplementary Figure S4) expressed *35S:AsTjYUC6* and exhibited self-compatibility (Figure 6). The degree of seed set following self-pollination was, however, lower than that of the T_0_-15 parental plant. Consistent with the T_0_ line phenotypes, again, no differences in pistil, filament length (Figure 7a–c), or vegetative characteristics were observed between the T_1_ plants and the wild-type S-morph.

**Figure 8:**
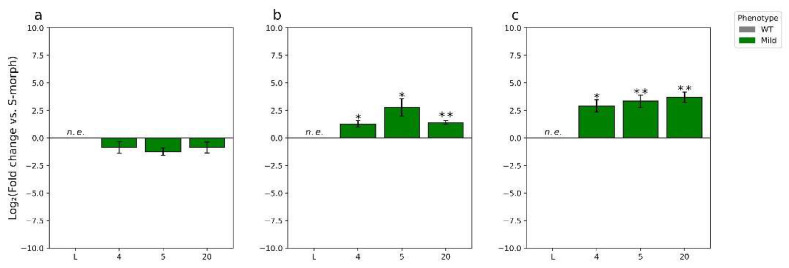
RT-qPCR analysis of the expression of TjYUC6 in T_1_ lines compared to WT S-morph during early floral development (**a**), late floral development (**b**), and the pollen from dehisced anthers (**c**). N = 3 biological replicates, n.e. = not expressed, * = *p*-value < 0.05, and ** = *p*-value < 0.01. *p*-values determined by Student’s *t*-test comparing the ∆CT of the respective transgenic line with that of the WT S-morph. Error bars represent the standard error of the fold change. Values represent the log2(fold change).

Hypothesizing that the reduction in SC in the T_1_ generation, relative to the T_0_ transgenic line, might be a result of transgene silencing (which is frequently associated with the 35S promoter [63]), leading to a reduction in repression of *TjYUC6*, we quantified the mRNA levels of *TjYUC6* in the T_1_ plants (Figure 8a–c). The reduction in the phenotype of the T_1_ generation was indeed found to be associated with a weaker degree of silencing; both the phenotype and the level of *YUC6* knockdown observed in these plants was similar to those of T_0_ plant T_0_-41. The expression patterns of *TjYUC6* through development observed in the T_1_ were similar to those observed for the primary transgenic plants; young anthers showed decreased expression compared to the S-morph (Figure 8a), while the mature anthers and pollen showed an increase in expression compared to the S-morph (Figure 8b,c). However, similar to the T_0_, the mature anther did not reach levels comparable to the WT S-morph’s young stamen (Figure S10a). When comparing expression levels across development, T_1_-4 and 20 showed expression patterns similar to T_0_-41 (Figure S10b,c). Like the T_0_ generation, the T_1_ generation’s pollen showed increased *TjYUC6* expression compared to the mature anthers (Figure S10d).

## 3. Discussion

### 3.1. TjYUC6 Is the Male Determinant Factor in Distylous Turnera

In distylous *Turnera*, *YUC6* was predicted to determine the male mating type [9], likely via indole-3-acetic acid (IAA) biosynthesis [64]. Here, we present empirical evidence that *YUC6* is the male determinant gene and establishes the mating type during early anther development. The knocking down expression of *TjYUC6* in S-morph plants led to their pollen being capable of fertilizing S-morph pistils, in both self- and cross-pollinations. Indeed, the line exhibiting the greatest knockdown (T_0_-15) had pollen that was rejected by L-morph pistils and showed a concomitant reduction in pollen size, suggesting that it had fully converted to the “L-morph” pollen mating type (Figure 1). Further, and importantly, the knockdown of *TjYUC6* had no impact on the pistil mating-type behavior. These results make *YUC6* the first male determinant gene to be functionally confirmed in any heterostylous species. *YUC6* homologs are important for male fertility in several species, including *A. thaliana* [64,65,66,67], *Fragaria vesca* [68], *Gossypium hirsutum* [69], *Zea mays* [70], and *Oryza sativa* [71,72]. The *Turnera S*-gene *YUC6* likely arose via the duplication of a progenitor that played a role in male fertility. After duplication, neofunctionalization somehow resulted in the copy in the *S*-locus evolving into the male determinant gene in heterostyly. What changes led to this neofunctionalization are not entirely clear. Our previous transcriptome analyses are consistent with the presence of *YUC6* leading to increased auxin production in anthers [21], and there are no obvious alterations in the amino acid sequence of *YUC6* relative to other YUCCA family members; however, we cannot rule out the possibility that *YUC6* might have modified or additional enzyme activity. Further, we did not identify any difference in the expression pattern of *Turnera YUC6* relative to *AtYUC6*, but additional study at the cellular level is needed.

That the SC phenotype of *AS-YUC6* transgenic plants was correlated with a decrease in the *TjYUC6* transcript level (relative to the WT S-morph), only during the early stages of anther development, strongly suggests that the male mating type is determined during a specific developmental window prior to, or very early in, microsporogenesis, well in advance of microgametogenesis. This is what would be predicted given the sporophytic nature of heteromorphic self-incompatibility (HSI) and a recent report of *AtYUC6* function in pollen development in *Arabidopsis* [67]. In *Arabidopsis*, *AtYUC6* is first expressed in the anther tissues and microsporocytes prior to the initiation of meiosis (stage 9), and continues to be expressed until stage 13 of pollen development when anthesis occurs (stage 13) [67,73]. *AtYUC6* activity is crucial to the very early stages of pollen development, as the pollen of *Atyuc6/Atyuc2* double knockout *Arabidopsis* mutants abort and degenerate prior to meiosis I, at the very beginning of microgametogenesis [67]. Though pollen development can be rescued by the expression of *AtYUC2* driven by a microspore-specific promoter (*proLat52*), it could not be rescued by expression driven by the expression of *AtYUC2* in the anther tapetum [67]. Further, this rescue was genetically sporophytic; a single copy was sufficient to rescue all of the pollen. Together, these results suggest that auxin transport from the anther tissues to the microspores, if it occurs, is not sufficient to promote pollen development and further that the critical location and time point of *YUC6/YUC2* action in pollen development is in diploid sporophytic microsporocytes [67]. As HSI also functions sporophytically (i.e., pollen phenotype is determined by both alleles of the diploid parent and not the pollen’s genotype), it is likely that *TjYUC6* is expressed during the equivalent stages in *Turnera*, suggesting that the key events determining the male mating type are established before microsporogenesis. While the stages of floral and pollen development have not been defined in *Turnera*, and the cellular location of *TjYUC6* has not been defined, pollen development shows high levels of conservation across dicots and monocots [74], thus, there is likely to be consistency between *Turnera* and *Arabidopsis*.

Interestingly, seed set with self-pollination was always lower than with the outcrosses. Similar results were observed in *Primula forbesii*; knockdowns of the female determinant gene *CYP734A50* resulted in individuals with altered mating types that set less seed when selfed than when outcrossed [14]. This may suggest an additional layer to SI, as wild heterostylous self-compatible populations of *Primula oreodoxa* [75], *Hedyotis acutangula* [76], *Narcissus albimarginatus* [77], and *Ophiorrhiza japonica* [78], to name a few species, show greater seed set when outcrossed than when selfed, or it may simply reflect inbreeding depression in these obligately outbreeding species. 

### 3.2. Is Neofunctionalization of YUC Homologs a Common Mechanism in Breeding Barrier Evolution?

The neofunctionalization of the *S*-gene *YUC6* is exciting, but perhaps not surprising. The basis of male sex in several diecious plants has been linked to auxin. In *Trichosanthes kiriloqii*, the expression of a homolog of *AtYUC6* is upregulated in male flowers relative to female flowers, which suggests it has a role in male sex determination [79]. In a differential expression analysis of *Coccinia grandis*, several genes related to auxin were upregulated in male flowers relative to female flowers [80]. This was also observed in male flowers of *Spinacia oleracea* relative to female flowers [81]. Interestingly, auxin-related genes are found upregulated in female flowers relative to male flowers in *Asparagus officinalis* [82]. Additionally, exogenous IAA treatment will masculinize *Humulus lupulus* and *Mercurialis annua*, but this can cause feminization in *Cannabis sativus*, *Silene pendula*, and *Opuntia stenopetala* [83]. This suggests that feminization could be a result of over saturating plants with IAA and inadvertently lead to the negative feedback of male-related pathways, as IAA is a negative regulator of IAA production pathways [64].

Beyond dioecy, auxin may play roles in other SI species. The pollen of the *Orchidaceae* contains large amounts of auxin and treatment with auxin can result in SC or partially SC plants [84]. Though the role of the *YUCCA* family has not been investigated in the *Orchidaceae*, the presence of high auxin levels in pollen and the *YUCCA* family’s involvement in auxin synthesis suggests that a *YUCCA* gene might be involved. Similar results have been observed in *Theobroma cacao*, when treated with NAA early during floral development loses SI [85]. Beyond breeding barriers, the neofunctionalization of *YUCCA* family genes has been reported in asexual reproduction. A homolog of *AtYUC2*, which shows redundancy with *AtYUC6* is involved in gemma cup formation and dormancy in *Marchantia polymorpha* [86].

Overall, the trend towards the alteration of auxin-related pathways and the potential neofunctionalization of *YUCCA* genes in other reproductive systems supports the notion that this clade of *YUCCA* family members and/or genes involved in the tryptophan-dependent auxin biosynthesis pathway, more broadly, may be common targets in the evolution of reproductive barriers.

### 3.3. RT-qPCR Analysis Reveals Expression of TjYUC6 in Mature Pollen

Previous semiquantitative RT-PCR analysis showed the expression of *YUC6* in distylous *Turnera subulata* anthers decreases over the course of development [9]. We quantified the expression of *TjYUC6* in the mature anthers of our T_0_ and T_1_ lines, anticipating lower expression levels than WT S-morph. Counterintuitively, the mature anthers of all lines exhibited a significant increase in the *TjYUC6* transcript relative to the S-morph. This raised the question of whether *TjYUC6*’s transcripts are present in S-morph pollen. 

RT-qPCR analysis confirmed that the *TjYUC6* transcript is present in the pollen of the WT S-morph at higher levels than those found in mature anthers. It is important to note, we cannot determine whether *TjYUC6* is actively transcribed in mature pollen or if the transcript is being sequestered and retained in mature pollen. As the S-morph is hemizygous for the *S*-locus, if *TjYUC6* is actively being transcribed, we would anticipate only 50% of the pollen carrying the *S*-locus to contain the *TjYUC6* transcript. In contrast, if it is a case of developmental priming, we would anticipate all pollen to contain the *TjYUC6* transcript. The presence of *TjYUC6* in the pollen may explain why we see an increase in the expression of *TjYUC6* in the transgenic lines during the later stages of development. Further investigation is necessary to resolve this question. 

### 3.4. YUC6 Acts Pleiotropically, Establishing Pollen Size Dimorphisms in Addition to Mating Type

In addition to changes in the male mating type, knocking down *TjYUC6* also led to a change in pollen size. Previous analyses of distylous species have characterized several traits that differ between the two morphs, including the anther placement, pistil length, and pollen grain size [87]. As *YUC6* is only expressed in anthers [9], it was unlikely to influence pistils [20]; however, it was unknown if the expression of this gene influences pollen size and/or filament phenotypes. Evidence from *Arabidopsis* has implicated *AtYUC6* in anther filament elongation [48] but not pollen wall formation [69]. 

In the most extreme transgenic lines, T_0_-15 and 63, we observed a significant reduction in pollen size, statistically similar to that of the L-morph. No other phenotypic changes were observed. A similar phenotype was observed in a self-compatible *T. joelii* mutant [53]. This suggests that *TjYUC6* acts pleiotropically to determine the pollen size in addition to the male mating type. As *Atyuc2/Atyuc6* double knockout mutants have irregularly shaped pollen [69], the over-production of auxin by *TjYUC6* in the S-morph may be establishing size dimorphisms. Alternatively, this may be an additional case of neofunctionalization. 

Minor changes in auxin levels have been shown to change phenotypes [35]. The lack of significant change in pollen size in transgenic lines T_0_-52 and 41, which had less knockdown of *TjYUC6* but still exhibited some change in the mating type, suggests there may be different auxin thresholds and/or mechanisms for determining the pollen size and male mating type, which is further supported by the finding that the establishment of the mating type is determined earlier (before the young, 4–6 mm bud stage) than the divergence in size was detected (after the young bud stage).

## 4. Methods

### 4.1. Plasmid Construction and Transformation

gDNA was isolated from ~0.1g of young buds (2–3 mm) from greenhouse-grown plants of *Turnera joelii* using Plant DNAzol (ThermoFisher, Waltham, MA, USA) and its accompanying protocol. The tissue was ground directly in DNAzol. See Table S1 for the primers used to amplify a 388bp fragment of *TjYUC6* (Supplementary Figures S1 and S2) that also contain added restriction sites to facilitate cloning. BLAST was used to confirm target specificity.

Accuzyme DNA polymerase (Bioline, Memphis, TN, USA) was used to amplify the fragment following the accompanying protocol. Restriction digests were performed using high-fidelity enzymes (Bam HI and Kpn I), and the CutSmart digest buffer (New England Biolabs, Ipswich, MA, USA). The *TjYUC6* fragment was ligated in reverse orientation behind the CaMV 35S promoter in pCHF3, a binary plasmid vector [63], using T4 ligase (Promega, Madison, WI, USA).

Plant transformation was performed, as previously described [88], with *Agrobacterium tumefaciens* (GV3101), with some minor modifications. Co-cultivation plates were incubated at room temperature in the dark for three days. Transformants were selected using media containing kanamycin. Shoots were transferred to new rooting media every month and maintained for approximately one year. After a year, any remaining shoots were dipped in Bontone II rooting powder (Bonide Products inc., Oriskany, NY, USA) and moved to soil. Greenhouse conditions were the following: 24–26 °C, day; 16–18 °C, night; and a 16 h photoperiod. Growth chamber conditions used for tissue culture were as follows: 27 °C and a 16 h photoperiod.

### 4.2. Genotyping

gDNA was isolated, as above, from putative transgenic lines. Quality was checked using primers designed for *β-tubulin* (Supplementary Table S1). The transformation of putative transgenic plants was confirmed using a CaMV 35S specific primer and the *AsTjYUC6* forward primer (as the insert was reverse orientation relative to the promoter). All PCR were performed using MyTaq 2× Red mix according to the manufacturer’s protocol (Bioline, Memphis, TN, USA). Both PCR and qPCR used an annealing temperature of 61.5 °C. PCR products were separated on 0.7% agarose gels for 30 min at 90 V and stained with ethidium bromide.

### 4.3. Quantification of Expression

RNA was isolated from young (4–6 mm) and mature (13–15 mm) anthers of the various transgenic lines and wild-type *T. joelii* using Concert Plant RNA Reagent following the accompanying protocol (Invitrogen, Carlsbad, CA, USA). RNA was isolated from the pollen of dehisced anthers using TRIzol (Thermo Fisher, Carlsbad, CA, USA) and a modified protocol (Supplementary Text S1). In both cases, tissue was ground directly in either the RNA reagent or TRIzol reagent. All RNA was treated with DNAse I (Thermoscientific, Carlsbad, CA, USA). cDNA was synthesized using SensiFast cDNA synthesis kit and the accompanying protocol (Bioline, UK). The amount of RNA and cDNA for all analyses were approximated using molecular ladders. Approximately 1µg of RNA and cDNA were used for related reactions, under the presumption that normalization with the housekeeping gene would compensate for any variation. SensiMix SYBR Low-ROX kit and the accompanying protocols were used for all RT-qPCR. Samples were run on an ABI 7500 fast machine (ABI, Vernon, CA, USA). Expression values were standardized using the housekeeping gene *β-tubulin*, which was amplified using previously described primers [9]. All primers can be found in Supplemental Table S1. RT-qPCR conditions can be found in Table S3.

### 4.4. Phenotyping

Screening for a change in mating type was performed for each line via the following crosses (Figure 1):

1. Transformants were initially self-pollinated and those that set seed considered self-compatible.
2. To confirm the retention of female mating type, transformants were crossed as pistil parents with the WT S- and L-morphs. Lines retaining the S-morph female mating type would only set seed with the L-morph.
3. To confirm the change in male mating type, the transformants were crossed as pollen parents with WT S- and L-morphs. Lines showing a change in male mating type would only set seed with the S-morph.

For each line, 5 anthers, 3 pistils, and 200 pollen grains were measured from 3 different flowers using ImageJ2 [89]. To quantify seed set, 10 replicates of the earlier described pollinations were performed for all transgenic lines and WT plants over a 30 day period. For figure purposes, the severity of phenotype refers to seed produced when a line was crossed with the L-morph compared to the amount of seed produced when a line was crossed with the S-morph. Severe refers to the inability to set seed with the L-morph; strong refers to the ability to set little seed (≤5 seed average) with the L-morph; moderate refers to the ability to set some seed (>5 seed average) with the L-morph; weak refers to the ability to set little seed (≤5 seed average) with the S-morph.

## 5. Concluding Remarks

Here, we characterized a novel role for a member of the *YUCCA* gene family. The *Turnera S*-gene *YUC6* is the first gene empirically shown to determine the male mating type and pollen size dimorphism. Our analyses support the previous hypothesis that the male mating type in *Turnera* is determined by altered auxin concentrations during microsporogenesis [9], and elucidates how a genetically hemizygous locus generates a genetically sporophytic HSI system in the S-morph of *Turnera*. 

## Data Availability

Not applicable.

## Supplementary Materials

The following supporting information can be downloaded at: https://www.mdpi.com/article/10.3390/plants11192640/s1, Table S1: primers sequences, Table S2: auxin-related genes that were previously identified as differentially expressed between the two morphs of *Turnera subulata*, Table S3: ABI fast 7500 conditions, Table S4: GenBank Accession number of *T. joelii* putative *YUC* homologs/, Figure S1: antisense *TjYUC6* oligonucleotide, Figure S2: diagram of the CDS of *TjYUC6*, Figure S3: alignment of *YUC6* with other YUCCA isoforms of *T. joelii*, Figure S4: confirmation of the presence of *As-TjYUC6* in transgenic T_0_ and T_1_ lines, Figure S5: expression of AS-YUC6 driven by the *35S* promoter in anthers and pollen of the transgenic line T_0_-15, Figure S6: comparison of the mature stamen of the WT S-morph and T_0_ lines with the young stamen of the WT S-morph, Figure S7: change in expression of *TjYUC6* over development, Figure S8: comparison of *AsTjYUC6* T_0_-15 with the WT S-morph, Figure S9: pollen throughout stamen development in the WT S-morph, Figure S10: change in expression of *TjYUC6* over development in the T_1_ lines, Supplemental Text S1: modified TRIzol protocol for RNA isolation from the pollen of *Turnera joelii*.
