# Supplementary material for: The S-Gene YUC6 Pleiotropically Determines Male Mating Type and Pollen Size in Heterostylous Turnera (Passifloraceae): A Novel Neofunctionalization of the YUCCA Gene Family
Source: Plants (Basel). 2022 Oct 8;11(19):2640. doi: 10.3390/plants11192640 (PMC9572539; doi:10.3390/plants11192640)
Supplement: Supplementary file 1 [file plants-11-02640-s001.zip › plants-1871745-supplementary.pdf]

Table S1: Primer sequences

Cloning

Gene	Forward Primer 5’ → 3’	Reverse Primer 5’ → 3’
TjYUC6 388bp insert + restriction sites (BAMHI and KPNI in bold)	**GGATCC**AACATACCCAACTAAAGACC	**GGTACC**ACAAGCGAAGGCTTAG

Genotyping

Gene	Forward Primer 5’ → 3’	Reverse Primer 5’ → 3’
β-tubulin	AGATTTTCCGACCCGACA	GCAATCACAATCTCGGCT
35S::AsTjYUC6	CCCACTATCCTTCGCAAGA	ACAAGAGCGGCGAGGA

RT-qPCR

Gene	Forward Primer 5’ → 3’	Reverse Primer 5’ → 3’
β-tubulin	AGATTTTCCGACCCGACA	GCAATCACAATCTCGGCT
WT *TjYUC6*	GATGGATTCCCTAGAGGCAC	CACCTTCCGATATCTTCTGCT
YUC2 (Tsub_00020676-RA)	GAACCAAGCAGCCGAGT	TCCATCCATTTGGGAATG
SAUR27 (Tsub_00014240-RA)	CAAAAGGGTTCTTGCCAGT	GTTGCTCATGACAGGGGA
GA20OX1 (Tsub_00006552-RA)	GAGGCCTGTGAAGAATATGCT	CAAAGAATGGTCAGGGCA
IPT5 (Tsub_00002732-RA)	GACACATCCACCCACAAGA	GAGGCCTGTGAAGAATATGCT
PIN6 (Tsub_00008196-RA)	TCAGCCCGGAGCAGT	AGCCCTCCACCGAAGA
CYP79A2 (Tsub_00006790-RA)	CTCTCCATCCTTGGCGT	CATGAACCCACCGGAA
35S::AsTjYUC6	CCCACTATCCTTCGCAAGA	TCTGCGCGTGAAACAGT

Primers were designed using the *Turnera subulata* transcriptome (GenBank: PRJNA589060)

Table S2: Auxin-related genes that were previously identified as differentially expressed between the two morphs of *T. subulata* [1]

**Turnera subulata**		**Arabidopsis thaliana homolog**		
**Transcript ID**	**Dev. Stage**	**Gene ID**	**Locus ID**	**Auxin related role**
Tsub_00006552-RA	Y	GA20OX1	At4g25420	Positively regulated by auxin, promotes cell expansion in the stalk of cabbage
Tsub_00002732-RA	Y	IPT5	At5g19040	Positively regulate auxin (IAA) biosynthesis
Tsub_00008196-RA	Y	PIN6	At1g77110	Promotes efflux of auxin
Tsub_00006790-RA	Y	CYP79A2	At5g05260	Involved in an alternative auxin biosynthesis pathway – synthesizes PAA
Tsub_00020676-RA	Y	YUC2	AT4G13260	Involved in the main tryptophane-dependent auxin biosynthesis pathway
Tsub_00014240-RA	M	SAUR27	At3g03840	Generally speaking, the SAUR family regulates growth in response to auxin

DE = differentially expressed. Y = 4-5mm buds, M = 13-15 mm buds. SvL – Comparison of expression in the S-morph vs L-morph, up indicates higher expression in the S-morph relative to the L-morph

Table S3: ABI fast 7500 conditions (used for all RT-qPCR)

**Stage**	**Temperature (ºC)**	**Duration (mins)**
Holding	**95.0**	**10:00**
Cycling (40 cycles)	95.0	00:15
	61.5	00:30
Melt Curve		
Step 1 (100%)	95.0	00:15
Step 2 (1%)	61.5	01:00
Step 3 (100%)	95.0	00:15
Step 4	61.5	00:15

Supplemental Table S4: *T. joelii* putative *YUC* homologs

**Transcript's name**	**GenBank accession number**
TjYUC6	OP354237
TJOE_DN40862	OP341804
TJOE_DN9991020999130511	OP341805
TJOE_DN99911591	OP341806
TJOE_DN20864	OP341807
TJOE_DN29995	OP341808
TJOE_DN882168599468157	OP341809
TJOE_DN99933987	OP341810
TJOE_DN21212	OP341811
TJOE_DN99916009987408	OP341812
TJOE_DN24925	OP341813
TJOE_DN8816959910190	OP341814
TJOE_DN8816009987407	OP341815
TJOE_DN9992428999367292	OP341816

Transcripts were pulled from previously published RNAseq data (PRJNA589060; Henning *et al.* 2020)

5’aacatacccaactaaagaccagtttattgcgtacgtggaggaatatgctaagaagttcgacatcaggccacacttcaatgagactgtttcacgcgcaga
gtatgatcaaaatctcgggttttggcacgtgaaaacgatagggctaatggcagaggagactgagtacgtgtgccggtgggtggttgtggctacagggga
gaatgcggaggcggtggtgccagagattgaagggatgagagagtttgttggagatataaggcatacaagtctgtacaagagcggcgaggactacaaa
ggcaaaaaggttttggtggtcggatgtgggaattcggggatggaggtctgcctggatctatgcaattataatgctaagccttcgcttgt

Figure S1 Antisense *TjYUC6* oligonucleotide

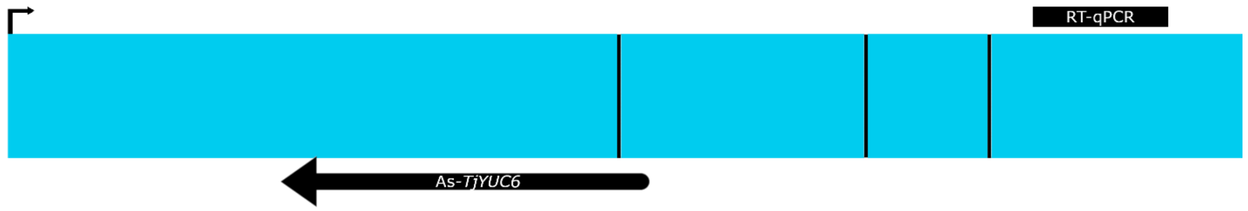

Figure S2: Diagram of the CDS of *TjYUC6*. Exons are separated by black lines. The fragment amplified via qPCR is indicated by the rectangle. The arrow indicates the region the antisense fragment compliments. The bent arrow represents the transcriptional start site.

Figure S3 Alignment of *YUC6* with other putative YUCCA isoforms of *T. joelii*

```
TJOE_DN20864                          TAACTTCCATGCCTGAATTACCACACCCAACAACCAGAACCCTCTGGTTCTTGAACACCG 879
TjYUC2                                AGACCTCCATGCCTGAATTCCCACACCCAACCACCAGCACCCTCTTCCCTCTGAATATTT 1031
TJOE_DN40862                          TGCCCTTCCCCCCTGAGCTACCCACATTCGTCCCTAGGGTTGGTTTCTGCAAC------T 588
TJOE_DN99916009987408                 TCCCCTTCCCCAAATCCTATCCACAATACGTCCCAAAAGAACAGTTCATAAAG------T 293
TJOE_DN24925                          TCCCCTTCCCCAAATCCTATCCACAATACGTCCCAAAAGAACAGTTCATAAAG------T 406
TJOE_DN8816959910190                  TCCCCTTCCCCAAATCCTATCCACAATACGTCCCAAAAGAACAGTTCATAAAG------T 406
TJOE_DN8816009987407                  TCCCCTTCCCCAAATCCTATCCACAATACGTCCCAAAAGAACAGTTCATAAAG------T 293
TJOE_DN9991020999130511               TCCCATTCCCTGAGGACTTCCCTGAGTACCCTACGAAGAGGCAGTTCATTGAC------T 534
TJOE_DN99933987                       TGCCTTTCCCAGAGGACTTCCCTGAATACCCAACCAAATTTCAGTTCATCACC------T 220
TJOE_DN21212                          TGCCTTTCCCAGAGGACTTCCCTGAATACCCAACCAAATTTCAGTTCATCACC------T 333
TJOE_DN99911591                       TTGGTTTCCCGGAGAATTTCCCCAAGTACCCTACCCGCCAACAATTCATCTCC------T 664
TJOE_DN882168599468157                TGGGATTCCCCAGCGATTTTCCAACCTACCCTACTAAAGACCAGTTCATCGGG------T 892
TJOE_DN29995                          TGGGATTCCCCAGCGATTTTCCAACCTACCCTACTAAAGACCAGTTTATCGGG------T 953
TjYUC6                                TGGGATTCCCAAGTGAATATCCAGCATACCCAACTAAAGACCAGTTTATTGCG------T 320
AsYUC6                                ------------------atccaacatacccaactaaagaccagtttattgcg------t 36
TsYUC6                                TGGGATTCCCAAGTGAATATCCAACATACCCAACTAAAGACCAGTTTATTGCG------T 319
                                                          **           *          *

TJOE_DN20864                          AACCGGTCTTGTACGCACTTGTATGCAAAATTTGACCATTGA---ACTGGTCAATACCAA 936
TjYUC2                                CTCCTGTCTTGTACGAGCTAGTGTGCATGATATGCCCACCAA---ACCCTTCCATTCCTT 1088
TJOE_DN40862                          ACCTGGACAATTATGTGTCTACTTTTGGGATAAAACCAAGGTGCAACAGGACTGTGGAGT 648
TJOE_DN99916009987408                 ACTTGGATGACTACGTTTCCCATTTCAAGATCACTCCTGTGTACCAGAGACGCGTGGAGC 353
TJOE_DN24925                          ACTTGGATGACTACGTTTCCCATTTCAAGATCACTCCTGTGTACCAGAGACGCGTGGAGC 466
TJOE_DN8816959910190                  ACTTGGATGACTACGTTTCCCATTTCAAGATCACTCCTGTGTACCAGAGACGCGTGGAGC 466
TJOE_DN8816009987407                  ACTTGGATGACTACGTTTCCCATTTCAAGATCACTCCTGTGTACCAGAGACGCGTGGAGC 353
TJOE_DN9991020999130511               ACCTTGAGTCCTACGCAAAGCAATTCGAGATCAACCCAAGGTTCAATGAGTGTGTCCAAT 594
TJOE_DN99933987                       ACCTTGAATCCTACGCACAACACTTCAACATAAACCCACATTTCAATGAGACAGTGCAGT 280
TJOE_DN21212                          ACCTTGAATCCTACGCACAACACTTCAACATAAACCCACATTTCAATGAGACAGTGCAGT 393
TJOE_DN99911591                       ACATGGAGTCCTACGCCTCCCACTTCTCAATCAAGCCTAGATTCGAGCAGGCAGTGCAAA 724
TJOE_DN882168599468157                ACTTGGAGGAATACGCTAGGAAGTTTGACATCAGGCCGCAGTTCAATGAGGCCGTGTCGC 952
TJOE_DN29995                          ACTTGGAGGAATACGCTAGGAAGTTTGACATCAGGCCACAGTTCAATGAGGCCGTGTCAC 1013
TjYUC6                                ACTTGGAGGAATATGCTAAGAAGTTCGACATCAGGCCACACTTCAATGAGACTGTTTCAC 380
AsYUC6                                acgtggaggaatatgctaagaagttcgacatcaggccacacttcaatgagactgtttcac 96
TsYUC6                                ACGTGGAGGAATATGCTAAGAAGTTCGACATCAGGCCACACTTCAATGAGACTGTTTCAC 379
                                           *     ** *        *     **    **        *        *

TJOE_DN20864                          GTATTTCAGGTATTACGGGCTCAGCATTCTCGCCTGTGGCAA------------------ 978
TjYUC2                                CGAATCGCGGCACAACTTCCTCTGCATTCTCCCCTGCGGCCA------------------ 1130
TJOE_DN40862                          TTGCATCCTACAATGAGAAGGATGAGAAGTGGCTTATTAGGGTTGAGAAT------ACTG 702
TJOE_DN99916009987408                 AGGCTGGTTTTGACGAAGAAACCAAGAAATGGAACGTTAAGGCAAGAAATGTGATCAG-- 411
TJOE_DN24925                          AGGCTGGTTTTGACGAAGAAACCAAGAAATGGAACGTTAAGGCAAGAAATGTGATCAG-- 524
TJOE_DN8816959910190                  AGGCTGGTTTTGACGAAGAAACCAAGAAATGGAACGTTAAGGCAAGAAATGTGATCAG-- 524
TJOE_DN8816009987407                  AGGCTGGTTTTGACGAAGAAACCAAGAAATGGAACGTTAAGGCAAGAAATGTGATCAG-- 411
TJOE_DN9991020999130511               CAGCTAGGTACGATGAGACCAGCGGCTTGTGGAGGGTCAAGACCGTCTCCGCAAGCGGCT 654
TJOE_DN99933987                       CTGCCAAGTACGACCAGACTTTTGGCCTGTGGCGAGTCAAAACCATCTCCATGAGCACTT 340
TJOE_DN21212                          CTGCCAAGTACGACCAGACTTTTGGCCTGTGGCGAGTCAAAACCATCTCCATGAGCACTT 453
TJOE_DN99911591                       GGGCTGAGTTTGATCACACGAGTGGGTTGTGGAAGGTAGAA------------------- 765
TJOE_DN882168599468157                ACGCGGAGTACGATCGAGTTCTTGGGTTCTGGCGCGTGAGGACGGTAGGG---------C 1003
TJOE_DN29995                          ACGCGGAGTACGATCGAGTTCTTGGGTTCTGGCGCGTGAGGACGGTAGGG---------C 1064
TjYUC6                                GCGCAGAGTATGATCAAAATCTCGGGGTTTGGCACGTGAAAACGATAGGG---------C 431
AsYUC6                                gcgcagagtatgatcaaaatctcgggttttggcacgtgaaaacgataggg---------c 147
TsYUC6                                GCGCAGAGTATGATCAAAATCTCGGGTTTTGGCACGTGAAAACGATAGGG---------C 430
                                                                   *

TJOE_DN20864                          -----CAATAAGCCACCTAGAAATGTACACGAGGTCTTGAGTT--------------TC 1018
TjYUC2                                -----CGACCAGCCATTGGCATGCATATTCGGTCTCTTCCAGCTTCAACCCCAGGGACTT 1185
TJOE_DN40862                          CCCTACATGAGTATGAAACTTACATGGCGAAGTTTCTTGTGGTTGCAACTGGGGAGAACA 762
TJOE_DN99916009987408                 -GTCTGGTGATATCGAGGACTACTCTGCCAGGTTTCTGGTGGTGGCAACGGGAGAAACAA 470
TJOE_DN24925                          -GTCTGGTGATATCGAGGACTACTCTGCCAGGTTTCTGGTGGTGGCAACGGGAGAAACAA 583
TJOE_DN8816959910190                  -GTCTGGTGATATCGAGGACTACTCTGCCAGGTTTCTGGTGGTGGCAACGGGAGAAACAA 583
TJOE_DN8816009987407                  -GTCTGGTGATATCGAGGACTACTCTGCCAGGTTTCTGGTGGTGGCAACGGGAGAAACAA 470
TJOE_DN9991020999130511               CCACCCGCACAGAGATGGAGTACATCTGCCGGTGGCTCGTCGTGGCCACCGGAGAGAATG 714
TJOE_DN99933987                       CACCTCCCACTGAGATAGAGTACATTTGCAAGTGGCTTGTGGTGGCCACCGGAGAGAATG 400
TJOE_DN21212                          CACCTCCCACTGAGATAGAGTACATTTGCAAGTGGCTTGTGGTGGCCACCGGAGAGAATG 513
TJOE_DN99911591                       -----ACTCAAGACCTCGTGTACATTTCTAGGTGGCTTATTGTTGCCACAGGCGAGAATG 820
TJOE_DN882168599468157                TGAAGGGAGAGGAGACGGAGTACGTGTGCCGGTGGCTGGTGGTGGCGACGGGGGAGAATG 1063
TJOE_DN29995                          TGAAGGGAGAGGAGACGGAGTACGTGTGCCGGTGGCTGGTGGTGGCGACGGGGGAGAATG 1124
TjYUC6                                TAAAGGAAGAGGAGACTGAGTACGTGTGCCGGTGGGTGGTTGTGGCTACAGGGGAGAATG 491
AsYUC6                                taatggcagaggagactgagtacgtgtgccggtgggtggttgtggctacaggggagaatg 207
TsYUC6                                TAATGGCAGAGGAGACTGAGTACGTGTGCCGGTGGGTGGTTGTGGCTACAGGGGAGAATG 490
                                                                          *

TJOE_DN20864                          TACCTTCCACAACCCACTCGTGTGATCAAATTCAGCCCTTTGCACTGCCTGCTCGAATTT 1078
TjYUC2                                GACCCGCCAGAACCCGCAGCAATGGTCGAACTCAGCGCTCACTACTGTGGTGTTGAAAAC 1245
TJOE_DN40862                          GTGAAGGAATCATTCCTGAGATCCCCGGA-TTAGACAGCTTTAAG------GGGACCTAC 815
TJOE_DN99916009987408                 CCGACCCTTCTGTCCCCGAGGTTGAAGGA-TTGGATTCTTTCACTGGTAGTGGAGAGGTT 529
TJOE_DN24925                          CCGACCCTTCTGTCCCCGAGGTTGAAGGA-TTGGATTCTTTCACTGGTAGTGGAGAGGTT 642
TJOE_DN8816959910190                  CCGACCCTTCTGTCCCCGAGGTTGAAGGA-TTGGATTCTTTCACTGGTAGTGGAGAGGTT 642
TJOE_DN8816009987407                  CCGACCCTTCTGTCCCCGAGGTTGAAGGA-TTGGATTCTTTCACTGGTAGTGGAGAGGTT 529
TJOE_DN9991020999130511               CAGAGTGTGTAATGCCCGAAATTGAAGGA-CTAAATGAGTTTGGC------GGTGATGTT 767
TJOE_DN99933987                       CAGAGAGGGTTGTGCCAGAGTTTGAAGGC-CTGCAAGATTTCGGT------GGCCCTGTA 453
TJOE_DN21212                          CAGAGAGGGTTGTGCCAGAGTTTGAAGGC-CTGCAAGATTTCGGT------GGCCCTGTA 566
TJOE_DN99911591                       CTGAGCCCGTAATACCTGAAATACTTGGT-ATTGACCAGTTCAAT------GGTCAAATT 873
TJOE_DN882168599468157                CGGAGGCGGTGGTGCCGGAGATTGAAGGG-ATGGGGGAGTTTGGT------GGGGATATA 1116
TJOE_DN29995                          CGGAGGCGGTGGTGCCGGAGATTGAAGGG-ATGGGGGAGTTTGGT------GGGGATATA 1177
TjYUC6                                CGGAGGCGGTGGTGCCAGAGATTGAAGGG-ATGAGAGAGTTTCTT------GGTGATATA 544
AsYUC6                                cggaggcggtggtgccagagattgaaggg-atgagagagtttgtt------ggagatata 260
TsYUC6                                CGGAGGCGGTGGTGCCAGAGATTGAAGGG-ATGAGAGAGTTTGTT------GGAGATATA 543
```

```
                                              *                *

TJOE_DN20864                             AGG--CTTGATTGAGAAGTGGAGGCATAGGAC-TCCATGTAGGAGATAAATTGTTGGCG 1135
TjYUC2                                   TGG--GTGTAGCTCGAAATGGTCCGAGTAAGAC-TCCAAGTAGGTCAAGAACTGGTGTTT 1302
TJOE_DN40862                             TTCCACTCAAATACGTACCAGAATGGAAAATGCTTTACTGGACAAAAG------------ 863
TJOE_DN99916009987408                    ATTCACTCTACCCAGTACAAAACGGGGAAGACCTACCAGGACATGAGTGTTTTGGTTGTT 589
TJOE_DN24925                             ATTCACTCTACCCAGTACAAAACGGGGAAGACCTACCAGGACAGGAGTGTTTTGGTTGTT 702
TJOE_DN8816959910190                     ATTCACTCTACCCAGTACAAAACGGGGAAGACCTACCAGGACAGGAGTGTTTTGGTTGTT 702
TJOE_DN8816009987407                     ATTCACTCTACCCAGTACAAAACGGGGAAGACCTACCAGGACATGAGTGTTTTGGTTGTT 589
TJOE_DN9991020999130511                  TTACACGCTTGCACTTACAAGTCCGGCGAGAAATTCAAGGGCAAGAAAGTACTAGTCGTG 827
TJOE_DN99933987                          ATGCATGCCTGTGACTACAAAACTGGTCAAGGCTTTCAGGGAAAGCACGTGCTTGTCGTT 513
TJOE_DN21212                             ATGCATGCCTGTGACTACAAAACTGGTCAAGGCTTTCAGGGAAAGCACGTGCTTGTCGTT 626
TJOE_DN99911591                          TTGCATACAAGTGCGTACAAGACCGGTTCGGTGTTCAAGAACCAGAGGTTCTGGTTGTT 933
TJOE_DN882168599468157                   AGGCATACAAGCCTTTACAAGAGCGGAGAGGATTACAGAGGGAAAAAAGTTTTGGTGGTG 1176
TJOE_DN29995                             AGGCATACAAGCCTTTACAAGAGCGGAGAGGATTACAGAGGGAAAAAAGTTTTGGTGGTG 1237
TjYUC6                                   AGGCATACAAGTCTGTACAAGAGCGGCGAGGACTACAAAGGCAAAAAGGTTTTGGTGGTG 604
AsYUC6                                   aggcatacaagtctgtacaagagcggcgaggactacaaaggcaaaaaggttttggtggtc 320
TsYUC6                                   AGGCATACAAGTCTGTACAAGAGCGGCGAGGACTACAAAGGCAAAAAGGTTTTGGTGGTC 603
                                                 *       *

TJOE_DN20864                             -GGTAGGGTACTTGGGGAAATT----CTCCGGGAAACCAAGAAGTGGTAGTTCGCAGAAT 1190
TjYUC2                                   -AGTTGGGTAGGTAGGGTAGTT----TTGTGGGAATGGCATGAGAGGGAGCTCGCAGAAC 1357
TJOE_DN40862                             ------------------------------------------------------------ 863
TJOE_DN99916009987408                    GGGTCTGGTAATTCTGGCATGGAAATTGCACTAGACCTTGCAAATCATGGTGCAAAAACA 649
TJOE_DN24925                             GGGTCTGGTAATTCTGGCATGGAAATTGCACTAGACCTTGCAAATCATGGTGCAAAAACA 762
TJOE_DN8816959910190                     GGGTCTGGTAATTCTGGCATGGAAATTGCACTAGACCTTGCAAATCATGGTGCAAAAACA 762
TJOE_DN8816009987407                     GGGTCTGGTAATTCTGGCATGGAAATTGCACTAGACCTTGCAAATCATGGTGCAAAAACA 649
TJOE_DN9991020999130511                  GGTTGTGGAAACTCTGGCATGGAAGTTTCACTAGACCTTTGCAACCACAATGCCCACCCA 887
TJOE_DN99933987                          GGCTGTGGCAATTCAGGCATGGAAGTTTCTCTTGACCTTTGCAATCATAATGCAAGCCCA 573
TJOE_DN21212                             GGCTGTGGCAATTCAGGCATGGAAGTTTCTCTTGACCTTTGCAATCATAATGCAAGCCCA 686
TJOE_DN99911591                          GGGTGTGGTAATTCAGGCATGGAAGTTAGCTTGGACCTGTGCAGGCACAATGCAATCCCT 993
TJOE_DN882168599468157                   GGGTGTGGGAATTCGGGAATGGAGGTGTGCTTGGACCTATGTAATCATAGCGCTAAGCCT 1236
TJOE_DN29995                             GGGTGTGGGAATTCGGGAATGGAGGTGTGCTTGGACCTATGTAATCATAGCGCTAAGCCT 1297
TjYUC6                                   GGATGTGGGAATTCGGGGATGGAGGTCTGCCTGGATCTATGCAATTATAATGCTAAGCCT 664
AsYUC6                                   ggatgtgggaattcggggatggaggtctgcctggatctatgcaattataatgctaagcct 380
TsYUC6                                   GGATGTGGGAATTCGGGGATGGAGGTCTGCCTGGATCTATGCAATTATAATGCTAAGCCT 663

TJOE_DN20864                             TGTTTGGGGAGGTGGAGCTTAAGGCGATCATAAGTTTTGTGTTGCCAAAGTGAAGCTATG 1250
TjYUC2                                   TGCTTTGGAAGATGGAGGCGAAGGCGATCGTAGGTTTTGAGCTGCCATAGAGAAGCTATG 1417
TJOE_DN40862                             ------------------------------------------------------------ 863
TJOE_DN99916009987408                    T-----CCATCGTTATTCGTAGCCCGATCCACATA-----TTGACGAGGGGCATGGTTTA 699
TJOE_DN24925                             T-----CCATCGTTATTCGTAGCCCGATCCACATA-----TTGACGAGGGGCATGGTTTA 812
TJOE_DN8816959910190                     T-----CCATCGTTATTCGTAGCCCGATCCACATA-----TTGACGAGGGGCATGGTTTA 812
TJOE_DN8816009987407                     T-----CCATCGTTATTCGTAGCCCGATCCACATA-----TTGACGAGGGGCATGGTTTA 699
TJOE_DN9991020999130511                  T-----CAATGGTGGTTCGCAGCTCGGTTCATGTT-----CTGCCGAGAGAAATATTTGG 937
TJOE_DN99933987                          T-----CAATGGTGGTTCGAAGCTCGGTCCATGTT-----TTGCCCAGAGAAGTCTTTGG 623
TJOE_DN21212                             T-----CAATGGTGGTTCGAAGCTCGGTCCATGTT-----TTGCCCAGAGAAGTCTTTGG 736
TJOE_DN99911591                          C-----ACATGGTTGTCAGAAACTCAGTACATGTT-----CTTCCAAGAGAGATGTTTGG 1043
TJOE_DN882168599468157                   T-----CACTCGTGGTTAGAGATTCGGTGCATGTT-----CTACCACGAGAGATGCTAGG 1286
TJOE_DN29995                             T-----CACTCGTGGTTAGAGATTCGGTAAGTATA-----TGTTCATATATACCTATATA 1347
TjYUC6                                   T-----CGCTTGTGGTAAAAGATAAAGTGCATGTT-----TTACCACAAGAGATGCTTGG 714
AsYUC6                                   t-----cgcttgt----------------------------------------------- 388
TsYUC6                                   T-----CGCTTGTGGTAAAAGATAAAGTGCATGTT-----TTACCACAAGAGATGCTTGG 713
```

Supplemental Figure S3: Clustal Omega alignment of putative *YUC* homologs in *Turnera joelii*. Putative YUC2's ID is Tjoe_DN9992428999367292Putative_TjYUC2. See supplementary table S4 above for Genbank ID accession numbers

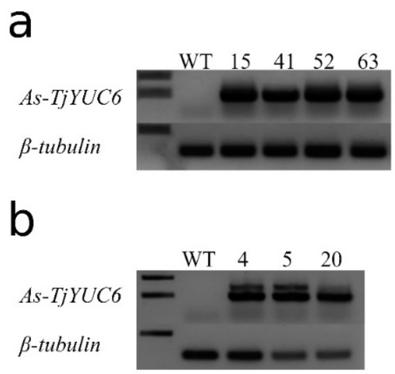

Figure S4: Confirmation of the presence of *As-TjYUC6* in transgenic T_0 (a) and T_1 (b) lines

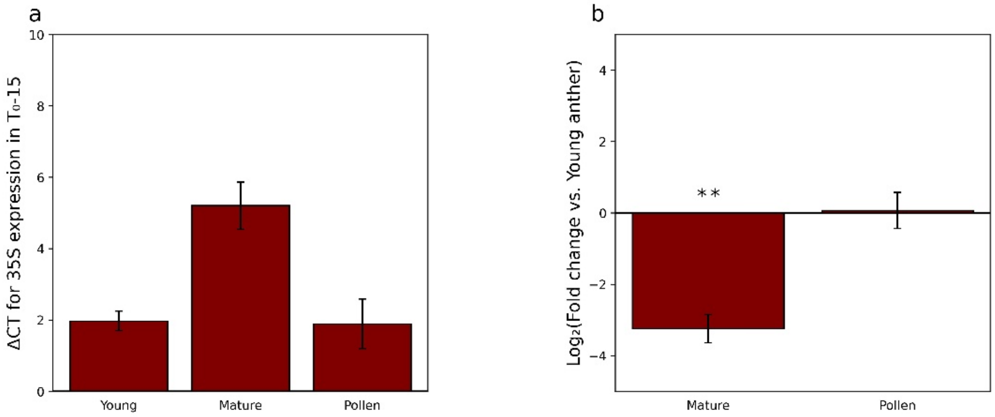

Figure S5: Expression of AS-YUC6 driven by the *35S* promoter in anthers and pollen of the transgenic line T_0-15. Normalized expression, shown as ΔCT (relative to expression of the housekeeping gene, ß-tubulin) (a), and comparison of relative expression of the transgene across anther development in T_0-15 (b). Note: ΔCT has an inverse relationship with expression level; the larger the ΔCT, the lower the expression of the gene relative to ß-tubulin. Compared with ß-tubulin, the 35S promoter driven construct showed decreased relative expression in the mature stage of anther development compared with the young anther stage. However, young anther and desiccated pollen exhibit approximately level of expression relative to ß-tubulin. Error bars represent the standard error. ** represents p-value < 0.01 as determined by Student's t-test. The data shown represent three biological replicates of each sample type.

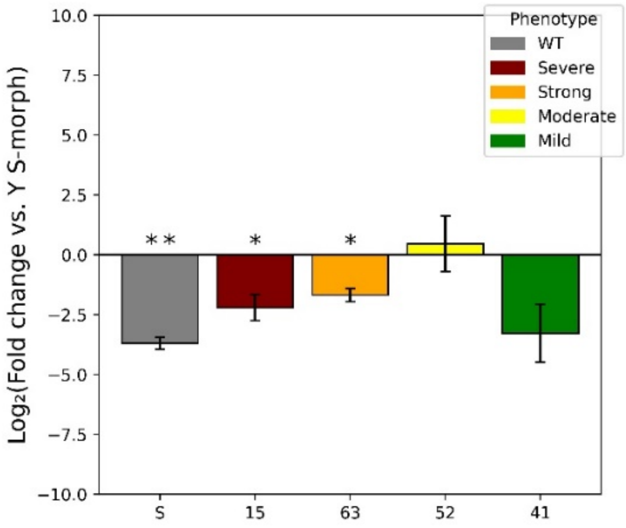

Figure S6: Comparison of expression of YUC6 in mature stamen of WT S-morph and T_0 lines, with WT S-morph young stamen. This analysis was used to determine if the increased expression of *TjYUC6* observed in the mature stamen of the transgenic lines ever reaches levels comparable to the levels observed in WT S-morph young stamen. N= 3 biological replicates. * = p-value < 0.05. ** = p-value < 0.01. p-values determined by Student's t-test. Error bars represent the standard error of the fold change. Values represent $\log_2$(fold change).

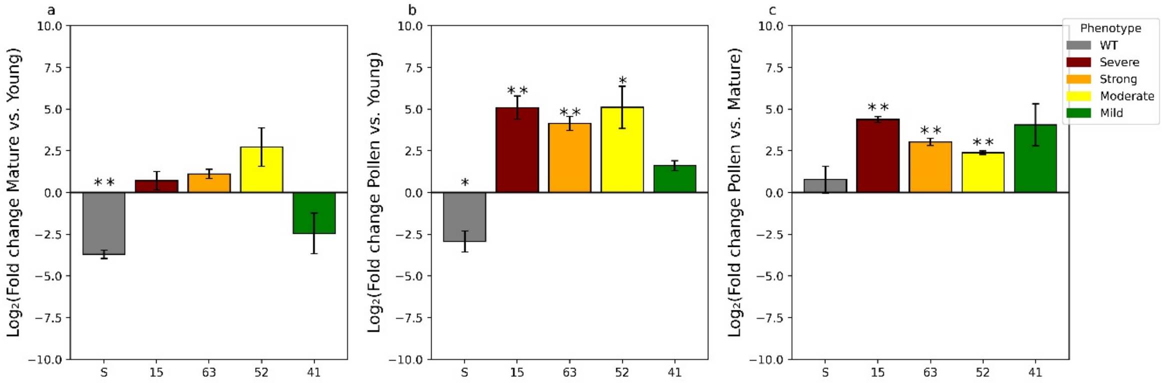

Figure S7: Change in expression of *TjYUC6* over development. Previous analysis showed a decrease in the expression levels of *TsYUC6* over the course of development [1,2]. Here we compare expression levels of *TjYUC6* in mature stamen with young stamen (a) and pollen with young stamen (b). To determine if the observed increase in expression of *TjYUC6* in the mature stamen of the transgenic lines may be due to expression of *TjYUC6* in developing pollen, we compared expression of *TjYUC6* in pollen with mature stamen (c). N = 3 biological replicates. * = p-value < 0.05, ** = p-value < 0.01. p-values determined by student's t-test. Error bars represent the standard error of the fold change. Values represent the $\log_2$(fold change).

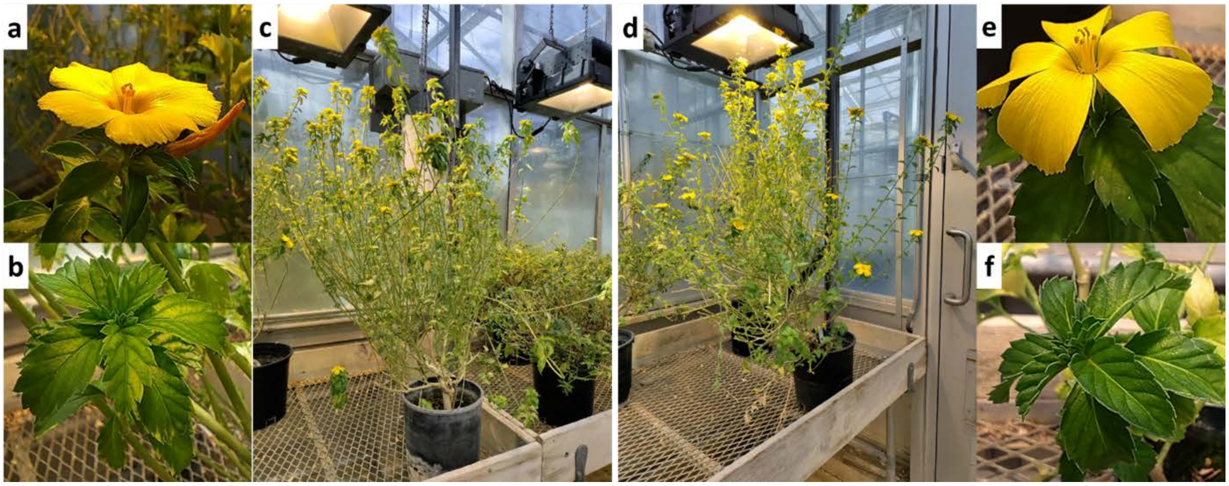

Figure S8: Qualitative comparison of *AsTjYUC6* T_0-15 (a-c) with the WT S-morph (d-f). Representative photos of flowers (a,e), leaves (b,f), and overall plant (c,d).

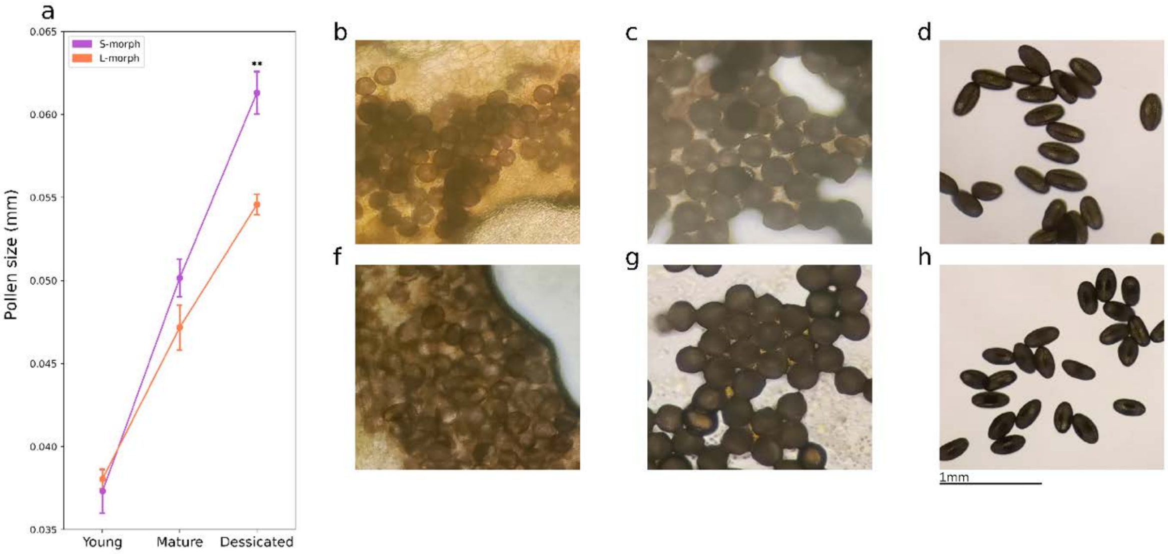

Figure S9: Pollen throughout stamen development in the WT S-morph. Range of pollen size throughout pollen development and post desiccation (a). n = 50 pollen from 3 flowers (total 150 pollen). Line graph represents the average pollen size, error bars represent the standard error. Only dry pollen size statistically differs between the two morphs following ANOVA $F_{5,12}$=48.1, $P<0.0001$ with an apriori contrast giving $F_{1,12}$ = 12.39 $P<0.004$ (denoted with **). The other stages do not differ significantly. Representative images of S- (b-d) and L-morph (f-h) pollen from young anthers (b,f), mature anthers (c,g), and desiccated anthers (d,h).

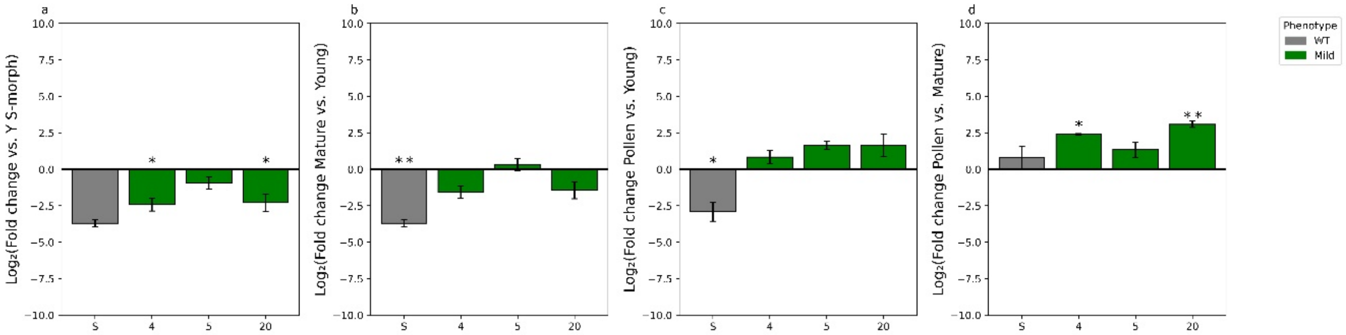

Figure S10: Change in expression of *TjYUC6* over development in the T_1 lines. Here we compare the expression levels of *TjYUC6* in the mature stamen with the WT S-morph's young stamen (a) and compare expression within lines (b-d) specifically of mature stamen vs young stamen (b), pollen with the young stamen (c), and pollen vs mature stamen(d). We see trends similar to those observed in T_0-41, which also showed a mild phenotype, confirming that T_1s' phenotypes are due to a decrease in *Yuc6* expression. N = 3 biological replicates. * = p-value < 0.05, ** = p-value < 0.01. p-values determined by student's t-test. Error bars represent the standard error of the fold change. Values represent the $\log_2$(fold change).

Text S1: Modified TRIzol protocol for RNA isolation from the pollen of *Turnera joelii*

1. Add the whole stamens of six flowers to 0.5 ml Trizol
2. Briefly vortex then centrifuge for 1 minute
3. Carefully transfer only pollen and Trizol into a new tube (leaving stamens and anthers in the tube)
4. Centrifuge for 1 minute
5. Grind resulting pellet, then vortex briefly
6. Incubate for 30 minutes
7. Centrifuge for 1 minute, move liquid to new tube
8. Add 0.1 ml of Chloroform
9. Incubate for 3 minutes
10. Centrifuge for 15 minutes
11. Transfer aqueous phase
12. Add 0.25 ml isopropanol
13. Incubate for 10 minutes
14. Centrifuge for 10 minutes
15. Discard supernatant
16. Resuspend pellet in 0.5 ml 75% ethanol
17. Vortex briefly then centrifuge for 5 minutes
18. Discard supernatant, pipette any remaining supernatant making sure not to disturb pellet
19. Air dry for 5 minutes
20. Resuspend in 20 µl of RNase free water

** All steps performed at room temperature

Citation

1. Henning P.M.; Shore. J.S.; McCubbin. A.G. Transcriptome and Network Analyses of Heterostyly in Turnera subulata Provide Mechanistic Insights: Are S-Loci a Red-Light for Pistil Elongation? *Plants*. **2020** 9(6): 713 https://doi.org/10.3390/plants9060713
